# Knowledge of Oral Hygiene among Hemophilic Patients Referred to Iranian Hemophilia Society

**DOI:** 10.5681/joddd.2009.014

**Published:** 2009-06-05

**Authors:** Mohammad Abrisham, Mehdi Tabrizizadeh, Alireza Ghateh

**Affiliations:** ^1^Dentist, Private Practice, Yazd, Iran; ^2^Associate Professor, Department of Endodontics, Faculty of Dentistry, Shahid Sadoughi University of Medical Sciences, Yazd, Iran; ^3^Assistant Professor, Department of Endodontics, Faculty of Dentistry, Shahid Sadoughi University of Medical Sciences, Yazd, Iran

**Keywords:** Bleeding disorders, hemophilia, knowledge, oral hygiene

## Abstract

**Background and aims:**

Hemophilic patients are faced with poor oral hygiene due to concerns about their dental care. The present study assessed the knowledge of hemophilic patients about oral hygiene and the effect of oral hygiene instruction in patients referred to Iranian Hemophilia Society.

**Materials and methods:**

This cross-sectional study was carried out on 30 hemophilic patients randomly selected from volunteer patients referred to Iran Hemophilia Center. The study was performed by means of a questionnaire submitted to subjects before and after the instructional brochure submission. The questionnaire included demographic data and items regarding hemophilia and oral hygiene. Data was analyzed with McNemar test and paired t-test.

**Results:**

The mean age of the patients was 21 years; 27 (90%) were males and 3 ones (10%) were females. They were mostly A hemophilia infected. Most patients enjoyed fair knowledge of oral hygiene. Changes in knowledge after reading the bro-chure were significant regarding the appropriate time to replace the toothbrush (P < 0.01), necessary visits for tooth examina-tions (P < 0.04), adjunctive methods of caries prevention (P < 0.001) and factors related to bleeding (P < 0.01); other factors improved slightly without significant changes.

**Conclusion:**

The knowledge of hemophilic patients was fair regarding oral hygiene while some relevant factors improved after instructions. However, more instruction is needed in order to attain more improvement in some behaviors.

## Introduction

Hemophilia is the most common congenital coagulation disorder with an incidence of 1:10000 persons.^1^ Hemophilia is an X chromosome-linked hereditary bleeding disorder (sex determining chromosome) due to a deficiency in blood coagulation factors. In this disease, blood coagulation factors are not produced in sufficient amounts and blood does not coagulate naturally. It is divided into hemophilia A (factor VIII deficiency) with an incidence of 1:10000, homophilia B (factor IX deficiency) with an incidence of 1:34000, and hemophilia C (factor XI deficiency) depending on deficiency of coagulation factors involved.^2,3^

Patient instruction in oral health is the responsibility of all the members of dental health care team. Oral health instruction develops in the patient an awareness of the need to return regularly for professional prophylaxis, examination and treatment. It helps patients recognize the preventive steps such as the use of fluoride. Appropriate dental care is necessary for all individuals; however, the issue gains more importance as dental care affects the general health of hemophilic patients.^4^ Confronted with specific complexities of the disease, the families with hemophilic patients do not pay sufficient attention to their dental care while this care plays an important role in the patients’ general health. The role of care is important in preventing invasive dental treatments. Due to the importance of successful dental treatments and prevention of possible risks, dental practitioners must be aware of infectious diseases like hepatitis and AIDS.

On the other hand, hemophilic patients are susceptible to dental caries and periodontal diseases more than ordinary people because of their inability to perform oral hygiene procedures.^3^ Although most dental procedures do not expose individuals to any risks, hemophilic patients are worried to receive dental treatments. For example, scaling, probing, use of mouthrinses, debridements, endodontic therapy, conventional restorations and prosthodontic procedures do not produce any risks for the patients.^5^ However, care must be taken not to inflict any trauma on gingival tissues. Some concerns have been raised in high-risk procedures such as the use of hypnosis for pain control,^6^ restorations without local anesthesia,^7^ use of interdental wedges before Cl II cavity preparations,^8^ and notch use with fissure bur in buccal and lingual surfaces during root canal therapy.^9^Adminstration of Amoxycillin 500 mg for 7 days post-surgically,^10,11^ or consultation with the physician involved must be considered for the treatment of high-risk hemophilic patients.^12^

Dental caries and periodontal diseases are two main oral diseases affecting all individuals, especially hemophilic patients. These two diseases are behavior-mediated, so they can be prevented by increasing knowledge of patients and cooperation of family and friends.

Due to a high incidence of hemophilia and since most patients with dental problems refuse to receive treatments for the fear of bleeding during the procedures,^13^ proper knowledge of the subject is necessary for these patients. Dentist awareness of the disease and its specific aspects are helpful in presenting treatment plans to prevent any possible risks. The present study evaluated the knowledge of hemophilic patients about oral hygiene and the effect of instructional brochures on improving their dental care level in patients referred to Iran Hemophilia Society in 2007.

## Materials and Methods

This cross-sectional study was conducted on 30 hemophilic patients referred to Iran Hemophilia Society in 2007. The patients were selected randomly on the condition that they gave their informed consent to participate in the study. The study questionnaire was given to the patients to obtain their knowledge in two phases: before and after reading the instructional brochure. The questionnaire included demographic data and questions about their knowledge, including the frequency of tooth brushing and frequency of dental visits a year. These questions were taken from the “Oral Health Impact Profile (OHIP)” with some modifications.^14^ The patients could choose more than one item in multiple choice questions. In the case when the patients were unable to answer the questions because of their age, their parents or guardians helped them.

The data obtained from the questionnaire was analyzed by means of SPSS Software, Version 13.0, and paired t and McNemar tests.

## Results

Thirty hemophilic patients participated in the study with a mean age of 21±11.47 years. Of all participants 27 (90%) were males and 3 (10%) were females. A total of 19 patients (67.9%) had hemophilia A, 7 patients (25%) had hemophilia B, and 1 patient (3.6%) was afflicted with hemophilia C. The severity of the disorder was slight in 6 patients (22.2%) and it was moderate in 8 patients (29.6%) and severe in 13 individuals (48.2%). Regarding treatment modalities, 23 patients (76.7%) received factor VIII, 3 patients (10%) received factor IX, 1 patient (3.3%) used blood products, and 1 patient (3.3%) received recombinant factor therapy. Two patients (6.7%) did not undergo any treatment modalities.

The disorder was under control in 26 subjects (92.9%). In patients over 16 years of age, 18 patients (85.7%) had high school education, and 3 patients (14.3%) said to have bachelor degrees. Furthermore, 20 patients (66.7%) completed the questionnaires themselves, 9 patients (30%) asked for help from their parents, and 1 patient (3.3%) asked his guardian to help him.

The results showed fair knowledge of hemophilic patients regarding oral hygiene as the frequency of patients correctly answering the questions before and after receiving instructional brochures was more than other options.

Statistical analysis showed no significant difference regarding patients’ knowledge about the methods used to clean the teeth, the frequency of daily tooth brushing, the use of dental floss, toothbrush type, toothpaste used to prevent caries, the effect of preventive procedures for hemophilic patients, the necessity of informing the dentist of the disease, necessary actions when bleeding occurred, and regarding gum bleeding a normal or abnormal condition before and after reading the brochure (P > 0.05). However, the patients’ knowledge regarding the time to replace their toothbrush, the necessary dental visits and adjunctive methods to prevent dental caries and the factors leading to bleeding were significantly different before and after the instruction (P < 0.05).

## Discussion

At present the role of community dentistry has broadened to prevent oral diseases among the public. Considering the high prevalence of hemophilia among people in different societies and the importance of education as a method to improve patients’ behavior, knowledge and acceptance of the existing treatment modalities, the present study evaluated hemophilic patients’ knowledge regarding oral hygiene and the improvement of their health care behavior after reading and instructional brochure.

The results of the present study showed improvements in patients’ knowledge and behavior in all the factors under study before and after reading the brochure, though some factors did not show statistically significant differences between the two phases. Significant differences were limited to factors of the time to replace the toothbrush, the factors leading to bleeding, and adjunctive methods used for the prevention of dental caries.

Hemophilia is a gender-related hereditary disorder observed in all societies.^15^ In our study, its prevalence was higher in males than in females (90% vs. 10%). According to statistics, more than 5000 hemophilic patients live in Iran and 3300 of them suffer from hemophilia A.^16^ These patients must receive blood products all their lives. Their specific characteristics, including fear of bleeding, focus on conventional treatment modalities, and inaccurate knowledge about suitable methods of dental services for hemophilic patients, prevent these patients from maintaining appropriate oral hygiene.

Pashapoor and Gol Mohammad Loo^16^ focused on the positive role of instruction in hemophilic patients; the results of the present study coincide with the results of that study.

There is no doubt that suitable medical and dental services are not available for all hemophilic patients in different societies,^13^ but most oral and tooth-related diseases can be prevented by increasing patients’ knowledge even with no access to suitable dental treatment modalities.

The results of the present study showed the success of instruction in increasing patient knowledge in some areas. This finding encourages the idea that improvement of oral hygiene in hemophilic patients is possible. Other educational methods including use of mass media in the field, multiple clinical appointments, home or school visits and vocational interventions can be effective in achieving better patient cooperation in decreasing possible risks of dental treatments and in presenting more preventive services. Further investigations are necessary to determine the role of different factors such as educational methods in knowledge of oral hygiene among the hemophilic patients.

## Conclusion

The results suggested that hemophilic patients have a fair knowledge regarding oral hygiene while some related factors can be improved after being instructed, although this improvement might not be significant in most cases.
